# Effect of rubber wood biochar on nutrition and growth of nursery plants of *Hevea brasiliensis* established in an Ultisol

**DOI:** 10.1186/2193-1801-1-84

**Published:** 2012-12-29

**Authors:** Randombage Saman Dharmakeerthi, Jayalath Arachchige Sarath Chandrasiri, Vishani Udayanga Edirimanne

**Affiliations:** Rubber Research Institute of Sri Lanka, Dartonfield, Agalawatta, 12200 Sri Lanka

**Keywords:** Biochar, *Hevea brasiliensis*, Nutrition, Plant growth, Rubber wood, Ultisol

## Abstract

Application of biochar alters availability of nutrients and acidic cations in soils which in turn could affect growth of plant to different degrees. Effect of rubber wood biochar amendment on the growth and nutritional status of *Hevea* nursery plants was determined in this study. Biochar were applied at 1% and 2% (w/w) with and without the recommended rates of N and Mg liquid fertilizers (LF). Two control treatments with 0% biochar but with and without recommended levels of all N, P, K, and Mg LF were also included. Application of biochar alone has a significant positive effect on above ground dry matter accumulation of the rootstock seedling (81% over the 0% biochar + no LF control) while no effect on the scion growth. Growth of plants in LF added treatments were much higher. Combining 2% biochar with N and Mg significantly increased the above ground dry matter accumulation over N-P-K-Mg only treatment in both rootstock seedling (29%) and the scion (61%). Biochar only application did not affect the N and P and decreased K and Ca concentrations in leaves. When combined with N and Mg fertilizers however, biochar significantly increased total N, P, Mg and Ca uptake. Biochar only application (2%) significantly decreased the leaf Mn concentrations in the seedling probably due to decrease in Mn availability as a result of increase in soil pH. The increase in soil pH due to biochar addition decreased with time close to original values in soils that received LF, possibly due to sulfate of ammonia. We concluded that application of rubber wood biochar (upto 2% w/w) could improve the growth of *Hevea* plants with the use of only N and Mg fertilizers under nursery conditions tested in this experiment.

## Background

Rubber *Hevea brasililiensis* L. (Willd. ex Adr. de Juss.) Müell. Arg.], a native tree spp. in the Amazon basin, was domesticated as a plantation crop in the south and southeast Asian countries during the latter part of the 1870’s. This commercially and environmentally important plantation crop has been now spread over 10.3 million hectares globally and it is dominated by the Asian region with 93% of the extent (International Rubber Study Group 2012). In the tropical Asian countries, rubber is grown on highly weathered soils characterized by very low organic C contents (Zhang et al. 2007) due to intensive cultivation over 100 years. Maintaining an appropriate level of soil organic matter and biological cycling of nutrients is crucial to the success of any soil management in the humid tropics. Application of compost, plant residues as mulching materials or growing cover crops have been adopted successfully to enhance nutrient cycling and use efficiency. However, such are experienced to be not sustainable technologies to enhance soil organic C reserves and maintain an improved soil fertility as most of organic matter decompose very rapidly under hot and humid tropical conditions (Jenkinson and Ayanaba 1977). Organic amendments, therefore, have to be applied repeatedly at short intervals to sustain soil productivity.

Conversion of biomass C into more stable biochar and amending degraded rubber growing soils with biochar appears to be an alternative technology to enhance fertility. Biochar is the biomass-derived char which was produced under more- and less-controlled conditions with and without total exclusion of oxygen and intended specifically for application to soil (Sohi et al. 2010). Physicochemical properties of biochar such as high porosity, high surface area, high charge density, high cation exchange capacity, high plant available nutrient contents and sometimes high pH associated with biochar have helped to improve fertility not only in degraded soils in the tropics (Glaser et al. 2002; Topoliantz et al. 2005; Steiner et al. 2007; Major et al. 2010) but also relatively more fertile soils in the temperate regions (Novak et al. 2009; Laird et al. 2010a) as well. In fact investigations on *Terra Preta de Indio* soils in the Amazon forest (Glaser et al. 2001; Lehmann et al. 2003; Liang et al. 2006), from where rubber plants were brought to the Asian continent, suggest that repeated applications of charcoal over a period of time could not only increase fertility in soils compared to those in non-charcoal added soils but also the effects could be long lasting.

Improvements in soil fertility after biochar application have lead to increased crop productivity. The magnitude of crop response however varied with the quality (Gaskin et al. 2010; Deenik et al. 2010) and quantity (Lehmann et al. 2003; Chan et al. 2007; Major et al. 2010) of biochar, soil type (Van Zwieten et al. 2010; Asai et al. 2009), plant species (Siregar 2007; Van Zwieten et al. 2010), other soil inputs (Lehmann et al. 2003; Steiner et al. 2007; Chan et al. 2008; Asai et al. 2009) or combination of these factors. Improvements in crop growth in biochar amended soils have often been attributed to increased nutrient availability resulted from high concentrations of plant available basic cations in biochar (Glaser et al. 2002; Uzoma et al. 2011), liming effect (Tryon 1948), changes in microbial community and enzyme activities (Rondon et al. 2007; Anderson et al. 2011; Chan and Xu 2009,2011), improved cation exchange capacity (Liang et al. 2006; Chan and Xu 2009), and also to improved moisture availability (Laird et al. 2010a).

However, application of biochar could sometimes negatively affect plant growth as observed by Kishimoto and Sugiura (1985) in yields of soybeans and maize with an addition of 5 Mg charcoal ha^-1^ and 15 Mg ha^-1^, respectively. Asai et al. (2009) observed that in nutrient poor soils in Laos, application of lumber mill waste biochar decreased the rice grain yield. Decreases in growth has been attributed to decreased N availability due to high C:N ratio in biochar (Lehmann et al. 2003; Rondon et al. 2007), or to the increased soil pH (Tryon 1948; Kishimoto and Sugiura 1985). Rubber plant is generally considered as a relatively insensitive species to pH, but the preferred soil pH ranges from 4.0 to 6.5 (Blackley 1997; Krishnakumar and Potty 1993; Priyadarshan 2003). Bolton (1964) considered the rubber plant as a “calciphobe”, growing badly in soils with pH 6.5 and over. Application of biochar could increase soil pH to levels greater than 6.5 immediately after application and lasts at least 1 pH unit higher than the native soil pH value even after several months (Dharmakeerthi et al. 2010; Uzoma et al. 2011; Van Zwieten et al. 2010; Chan et al. 2007).

Further, there is a strong antagonism between K and Mg uptake by rubber plants (Weerasuriya and Yogaratnam 1989; Singh et al. 2005) and application of biochar could alter the balance between the availability of the two nutrients in the soil. It has been observed that application of 1% (w/w) timber mill waste charcoal, made from hardwood at low temperatures using pit kiln technique, significantly decreased the dry matter accumulation of rubber seedling plants even when combined with 50% recommended N-P-K-Mg fertilizers (Dharmakeerthi et al. 2010). Moreover, application of timber mill waste charcoal increased K concentration and reduced Mg and Mn concentrations in leaves of rubber seedlings. Therefore, application of biochar could affect nutritional status and growth of the rubber plant depending on the pH buffer capacity of the rubber growing soils and other fertilizers applied.

Only a few studies have been conducted to investigate the effect of biochar application on woody plant species. Chidumayo (1994) reported better seed germination, shoot heights, and biomass production among native woody plants on soils under charcoal kilns relative to plants growth on undisturbed Zambian Alfisols and Ultisols. Siregar (2007) observed that the growth of two industrial forest plant species raised in poly-bags using sub-soils of an Ultisol from Java increased by about 100% when the soil was amended with charcoal made from wastes in woody forests and without any chemical fertilizer. A positive growth response from woody forest plants to charcoal application under field conditions have also been observed by Kishimoto and Sugiura (1985) and Ishii and Kadoya (1994). Published literature on the effect of biochar application on the growth of rubber plants, except for a study carried out in Sri Lanka using timber mill waste charcoal and rubber nursery plants established in an acidic Ultisol low in Mn Dharmakeerthi et al. (2010), is rather meager.

Biochar can be produced from any biomass material available. Many raw rubber manufacturing factories in the world use rubber wood as the energy source for their furnaces. Part of this firewood, otherwise burnt into ashes, could be converted to biochar by introducing appropriate technologies. We hypothesized in this study that biochar produced using rubber wood could be applied to rubber growing soils without affecting the growth of the rubber plant with judicious application of chemical fertilizers. Our objectives are (i) to determine the nutritional and growth responses of rubber nursery plants to rubber wood biochar when applied with and without chemical fertilizers, (ii) to identify possible causes for the responses in order to formulate appropriate fertilizer practices when biochar is used as a soil amendment in rubber nurseries. To achieve these objectives an experiment was conducted in a rubber nursery established under field conditions. Rubber plants were raised in polyethelene bags filled with an acidic Ultisol and the experiment spanned over the entire nursery period until plants were ready for field planting.

## Results

### Soil and biochar characteristics

The soil used was an acidic sandy loam with very low cation exchange capacity of 2.34 cmol(+) kg^-1^ and organic C content of 6.7 g kg^-1^. The available nutrient contents of the soil were to be judged as very low (Table 1). In contrast the rubber wood biochar used were highly alkaline showing a pH of 9.59 and had very high contents of available P, K, Mg and Ca (Table 1). Exchangeable Mg content was comparatively lower than exchangeable K and Ca contents. Cation exchange capacity of the biochar was also high as 13.87 cmol(+) kg^-1^.

**Table 1:** **Some relevant properties of the soil and biochar used**

Property	Unit	Soil	Biochar
Sand	g kg^-1^	800	-
Silt	g kg^-1^	34	-
Clay	g kg^-1^	166	-
pH (1:2.5 or 1:20 water)		4.37	9.59
Cation exchange capacity (1 *M* NH_4_OAc, pH 7)	cmol(+) kg^-1^	2.34	13.87
Organic C (Walkley & Black)	g kg^-1^	6.7	nd^*^
Ash content (ASTM D2974 1988)	g kg^-1^	963	53
Available P (1 M NH4F/0.5 M HCl, pH 1.8)	mg kg^-1^	8	747
Exchangeable K (1 M NH_4_OAc, pH 7)	mg kg^-1^	51	6895
Exchangeable Mg (1 M NH_4_OAc, pH 7)	mg kg^-1^	13	908
Exchangeable Ca (1 M NH_4_OAc, pH 7)	mg kg^-1^	159	9799
Total N (Se/H_2_SO_4_ + Na_2_SO_4_)	g kg^-1^	1.19	5.1
Total P (Se/H_2_SO_4_ + Na_2_SO_4_)	g kg^-1^	0.423	1.3
Total K (Se/H_2_SO_4_ + Na_2_SO_4_)	g kg^-1^	0.998	9.1
Total Mg (Se/H_2_SO_4_ + Na_2_SO_4_)	g kg^-1^	0.0225	4.3
Total Ca (Se/H_2_SO_4_ + Na_2_SO_4_)	g kg^-1^	0.0395	14.9

* nd – not determined.

### Growth of root stock seedling

Application of biochar without LF did not affect the leaf dry matter content compared to the 0% biochar-no LF control (Table 2). When the biochar rate was increased up to 2% without LF, a significant increase in the stem dry matter (5.8 g plant^-1^) as well as total shoot dry matter (9.4 g plant^-1^) could be observed compared to the 2.8 and 5.2 g plant^-1^, respectively of the 0% biochar-no LF control. The increase in the shoot dry matter due to the addition of 2% biochar was 81%. However, the increase in dry matter due to biochar without LF was significantly lower than those of LF applied treatments, either with or without biochar. When plants were supplied only with N-P-K-Mg at recommended rates, shoot dry matter content was 20.0 g plant^-1^ and was not significantly different from 1% biochar + N-Mg (21.3 g plant^-1^) treatment. A significantly higher dry matter accumulation in leaves, stems and shoots, compared to the 0% biochar + N-P-K-Mg or 1% biochar + N-Mg, could be observed in the plants of the 2% biochar + N-Mg treatment. The increase in shoot dry matter content in plants of the 2% biochar + N-Mg treatment over the current N-P-K-Mg application without biochar was 29%.

**Table 2:** **Mean** (± **standard deviation**) **values of dry matter accumulation in different components of the root stock seedlings at cut**-**back stage of 18 weeks after planting and the scion plants at 12 weeks after cut**-**back of the root stock seedling**

	Stock			Scion						
	Above ground			Above ground			Below ground			Root/Shoot ratio
Treatment	Leaf	Stem	Shoot	Leaf	Stem	Shoot	Tap root	Feeder + lateral root	Root	
	g plant^-1^									
0% biochar + no LF	2.3 ± 0.8^c^	2.8 ± 0.4^d^	5.2 ± 0.9^d^	1.2 ± 0.2^c^	1.3 ± 0.9^c^	2.5 ± 0.8^c^	6.1 ± 2.5^bc^	1.6 ± 0.5^b^	7.7 ± 2.5^b^	3.36 ± 0.28^a^
0% biochar + N-P-K-Mg	7.4 ± 1.8^b^	12.5 ± 2.9^b^	20.0 ± 4.0^b^	4.5 ± 1.9^b^	3.4 ± 1.4^b^	7.9 ± 1.4^b^	7.7 ± 1.6^ab^	3.9 ± 1.1^a^	11.6 ± 2.4^a^	1.61 ± 0.17^bcd^
1% biochar + no LF	2.9 ± 0.5^c^	4.4 ± 0.9^cd^	7.4 ± 1.1^cd^	1.8 ± 1.3^c^	1.4 ± 1.1^c^	3.2 ± 1.1^c^	5.4 ± 1.5^bc^	1.3 ± 0.7^b^	6.6 ± 1.7^b^	2.65 ± 0.24^abc^
1% biochar + N-Mg	8.7 ± 2.4^b^	12.6 ± 3.0^b^	21.3 ± 4.3^b^	7.2 ± 1.9^a^	4.5 ± 1.2^ab^	11.7 ± 1.2^a^	6.2 ± 1.5^bc^	5.0 ± 1.2^a^	11.2 ± 1.4^a^	1.00 ± 0.06^d^
2% biochar + no LF	3.6 ± 0.9^c^	5.8 ± 1.9^c^	9.4 ± 2.6^c^	1.3 ± 0.3^c^	1.2 ± 0.5^c^	2.4 ± 0.5^c^	4.7 ± 2.4^c^	1.6 ± 0.9^b^	6.3 ± 2.4^b^	2.86 ± 0.30^ab^
2% biochar + N-Mg	10.5 ± 1.8^a^	15.3 ± 3.4^a^	25.8 ± 4.4^a^	7.2 ± 2.3^a^	5.5 ± 0.6^a^	12.7 ± 0.6^a^	9.6 ± 1.9^a^	4.5 ± 1.4^a^	14.2 ± 2.8^a^	1.17 ± 0.19^cd^

Values followed by the same superscripts in a column are not significantly different at p < 0.05.

### Growth of grafted scion plant

Effect of biochar amendment on the growth of the scion was not as promising as in the seedling plant. Increasing the rate of biochar incorporation from 0 to 2% without LF did not have a significant influence on dry matter accumulation in leaves, stems or total scions. However, scion dry matter increased with increasing biochar level in the LF supplied treatments. A significant increase in dry matter content (61%) was observed in 2% biochar + N-Mg treatment over the currently recommended fertilizer application.

Most of the below ground parts of the grafted plants were consisted of tap roots of the seedling root stocks. These were about 77% and 64% in plants with and without LF, respectively. Mean tap root weight varied from 4.7 (in 2% biochar + no LF) to 9.6 g plant^-1^ (in 2% biochar + N-Mg) among treatments. Application of biochar did not influence the taproot weight in no LF treatments but, application of N-Mg with 2% biochar significantly increased the tap root weight (9.6 g plant^-1^) compared to all other treatments except for N-P-K-Mg without biochar treatment (7.7 g plant^-1^). The lateral cum feeder root weights and the total root dry matter in this experiment were significantly high in LF added treatments compared to the no LF treatments irrespective of the biochar levels. The root/shoot ratio ranged from 1.00 to 3.36 among treatments and was significantly low in chemical fertilizer added treatments.

### Nutrient status of the plant

Leaf P and Fe concentrations in both seedlings and scions and Zn in seedlings were not affected by the treatment (Table 3). Leaf N in seedlings was significantly high (2.53%) in 2% biochar + N-Mg treatment compared to that in the 0% biochar + no LF (1.74%). There was no significant difference in leaf N between LF added and no LF added counterparts at all biochar levels. Leaf N in the scion was significantly higher in LF added treatments than their no LF added counterparts. Increasing biochar level from 0 to 2% with or without LF did not affect leaf N concentrations significantly. However, in the no chemical fertilizer treatments, leaf N concentrations increased from 1.74 to 2.19% in the seedlings and from 2.60 to 2.93% in the scions, when biochar level was increased from 0 to 2%.

**Table 3:** **Mean** ( ± **standard deviation**) **values of leaf nutrient concentrations of the root stock seedlings at cut**-**back stage of 18 weeks after planting and the scion plants at 12 weeks after cut**-**back of the root stock seedlings**

Treatment	N	P	K	Mg	Ca	Mn	Zn	Fe
	%					mg kg^-1^		
	Seedling leaves							
0% biochar + no LF	1.74 ± 0.13^b^	0.18 ± 0.05^a^	1.27 ± 0.04^a^	0.16 ± 0.02^bc^	0.34 ± 0.01^a^	73 ± 19^b^	36 ± 13^a^	226 ± 38^a^
0% biochar + N-P-K-Mg	2.27 ± 0.27^ab^	0.19 ± 0.03^a^	0.71 ± 0.09^b^	0.24 ± 0.02^a^	0.22 ± 0.06^b^	138 ± 48^a^	32 ± 2^a^	185 ± 79^a^
1% biochar + no LF	2.12 ± 0.14^ab^	0.19 ± 0.04^a^	0.94 ± 0.34^b^	0.14 ± 0.03^c^	0.25 ± 0.03^b^	65 ± 25^b^	29 ± 15^a^	262 ± 98^a^
1% biochar + N-Mg	2.30 ± 0.51^ab^	0.20 ± 0.06^a^	0.45 ± 0.26^c^	0.22 ± 0.10^a^	0.15 ± 0.05^c^	66 ± 29^b^	32 ± 11^a^	188 ± 33^a^
2% biochar + no LF	2.19 ± 0.28^ab^	0.22 ± 0.04^a^	0.87 ± 0.13^b^	0.13 ± 0.03^c^	0.13 ± 0.03^c^	9 ± 1^c^	18 ± 5^a^	231 ± 76^a^
2% biochar + N-Mg	2.53 ± 0.20^a^	0.23 ± 0.02^a^	0.44 ± 0.08^c^	0.21 ± 0.04^ab^	0.12 ± 0.02^c^	102 ± 29^ab^	29 ± 15^a^	254 ± 97^a^
	**Scion leaves**							
0% biochar + no LF	2.60 ± 0.46^d^	0.27 ± 0.01^a^	1.24 ± 0.04^a^	0.39 ± 0.03^a^	0.58 ± 0.09^a^	47 ± 9^e^	34 ± 5^d^	196 ± 42^a^
0% biochar + N-P-K-Mg	3.92 ± 0.58^a^	0.29 ± 0.01^a^	1.12 ± 0.06^a^	0.30 ± 0.08^b^	0.30 ± 0.03^c^	91 ± 27^b^	69 ± 19^ab^	174 ± 71^a^
1% biochar + no LF	2.87 ± 0.35^cd^	0.31 ± 0.03^a^	1.20 ± 0.25^a^	0.20 ± 0.01^c^	0.39 ± 0.05^bc^	74 ± 12^cd^	57 ± 7^bc^	306 ± 139^a^
1% biochar + N-Mg	3.80 ± 0.26^ab^	0.30 ± 0.02^a^	0.68 ± 0.12^b^	0.21 ± 0.02^c^	0.46 ± 0.04^b^	127 ± 13^a^	78 ± 15^a^	213 ± 125^a^
2% biochar + no LF	2.93 ± 0.59^bcd^	0.28 ± 0.01^a^	1.07 ± 0.18^a^	0.22 ± 0.01^c^	0.42 ± 0.05^b^	68 ± 4^d^	48 ± 2^cd^	311 ± 85^a^
2% biochar + N-Mg	3.73 ± 0.50^abc^	0.30 ± 0.03^a^	0.79 ± 0.12^b^	0.20 ± 0.01^c^	0.41 ± 0.03^b^	86 ± 7^bc^	66 ± 6^ab^	276 ± 42^a^

Values followed by the same superscripts in a column at a given growth stage are not significantly different at p < 0.05.

Leaf K concentration was highest in the 0% biochar + no LF control, 1.27% in the seedling and 1.24% in the scion. As biochar level increased without LF, the K level decreased to 0.87% significantly in the seedling and to 1.07% in the scion at 2% biochar. In the LF added treatments, K was highest in the currently adopted 0% biochar + N-P-K-Mg treatment (0.71% in the seedling and 1.20% in the scion). Again, when biochar level increased with N-Mg, leaf K level decreased significantly, to 0.44% in the seedling and 0.79% in the scion at 2% biochar.

Magnesium concentration in the leaves of the seedling was significantly high in LF applied treatments compared to no LF treatments (Table 3). These concentrations were not influenced by the rate of biochar application. In the scion however, the highest Mg concentration (0.39%) was observed in the 0% biochar + no LF control treatment which was significantly higher than all other treatments. The next highest Mg concentration of 0.30% was observed in the 0% biochar + N-P-K-Mg treatment. Biochar application further decreased the leaf Mg concentration in the scion, either with or without Mg fertilizer.

Leaf Ca concentrations in the seedling and scion plants were high in the 0% biochar + no LF control (0.34 and 0.58%, respectively) compared to those in the currently adopted 0% biochar + N-P-K-Mg treatment (0.22 and 0.30% respectively). Application of biochar showed contrasting trends in the seedling and scion. In the seedling plants, leaf Ca concentrations decreased with increasing biochar rates, in both LF and no LF treatments. Besides, they were significantly high in no LF treatments except at 2% biochar rate where leaf Ca concentrations were comparable. In the scion, leaf Ca concentrations were significantly high in the biochar + N-Mg treatments compared to that in 0% biochar + N-P-K-Mg treatment. In no LF treatments it decreased significantly to 0.39% at 1%biochar and 0.42% at 2% biochar from 0.58% in the 0% biochar + no LF control.

Manganese concentrations were significantly higher in LF applied treatments than in their no LF counterparts in both seedlings and scions except for 1% biochar treatment of the seedling plants where they were comparable. In no LF treatments in the seedling plants, leaf Mn concentration decreased significantly with the increase in the biochar rate from 73 μg g^-1^ in the 0% biochar to 9 μg g^-1^ in the 2% biochar treatment. Such a trend was not observed in LF applied treatments in seedlings or any biochar applied treatments in the scion.

In no LF treatments, leaf Zn concentrations in the seedling decreased significantly from 36 μg g^-1^ in the 0% biochar + to 18 μg g^-1^ in the 2% biochar treatment. Leaf Zn concentrations in the LF received seedling plants were not significantly different and comparable to the levels of the 0% biochar + no LF control. In the scion however, leaf Zn concentrations were significantly high in LF added treatments compared to their no LF added counterparts. In LF added treatments, incorporation of biochar did not influence the scion leaf Zn levels significantly.

### Influences on soil properties

Some relevant chemical properties were determined in soil samples at bag filling and at the end of the experiment. Since LF application were initiated only one month after seed transplanting, soil properties at bag filling stage in LF added treatments were assumed to be the same as those of no LF added treatments. Mixing of 50 g compost and 50 g phosphate rock at bag filling stage has increased the soil pH from 4.37 to 5.30 (Tables 1 and 4). Mixing these soils with 1% and 2% biochar has further increased the pH upto 6.25 and 6.58, respectively (Table 4). Similarly exchangeable K, Mg and Ca levels also increased with biochar application. Available P levels were not significantly different at bag filling stage.

**Table 4:** **Mean** (± **standard deviation**) **values of some important soil properties measured at bag filling stage and at the end of the experiment**

	pH		Avail.P		Exch.K		Exch.Mg		Exch.Ca	
Treatment			mg kg^-1^							
	Bag filling	End	Bag filling	End	Bag filling	End	Bag filling	End	Bag filling	End
0% biochar + no LF	5.30 ± 0.01^c^	5.64 ± 0.11^a^	1699 ± 298^a^	1549 ± 85^a^	78 ± 34^c^	30 ± 9^cd^	22 ± 2^c^	14 ± 0^c^	248 ± 16^c^	220 ± 15^b^
0% biochar + N-P-K-Mg	-	4.31 ± 0.05^b^	-	1650 ± 222^a^	-	136 ± 7^a^	-	35 ± 1^a^	-	78 ± 5^d^
1% biochar + no LF	6.25 ± 0.03^b^	5.69 ± 0.09^a^	1570 ± 411^a^	1384 ± 297^a^	129 ± 41^b^	47 ± 12^c^	34 ± 6^b^	16 ± 2^c^	337 ± 31^b^	230 ± 22^b^
1% biochar + N-Mg	-	4.33 ± 0.08^b^	-	1585 ± 388^a^	-	17 ± 4^cd^	-	36 ± 6^a^	-	117 ± 16^c^
2% biochar + no LF	6.58 ± 0.01^a^	5.57 ± 0.33^a^	1474 ± 143^a^	1626 ± 198^a^	160 ± 48^a^	94 ± 42^b^	40 ± 4^a^	29 ± 2^b^	379 ± 28^a^	319 ± 18^a^
2% biochar + N-Mg	-	4.43 ± 0.11^b^	-	1535 ± 154^a^	-	12 ± 4^d^	-	41 ± 4^a^	-	136 ± 7^c^

Values followed by the same superscripts in a column are not significantly different at p < 0.05.

At the end of 8-month experimental period, soils without LF had pH values over 1 unit higher than their LF added counterparts. Although application of biochar increased the soil pH from 5.30 to 6.58 immediately, with time, the soil pH decreased on average to 5.60 in no LF treatments and to 4.36 in LF added treatments (Table 4). There was no significant effect on pH due to biochar addition at the end of the experiment, but in soils of LF applied treatments, pH values were significantly low compared to the no LF treatments. The exchangeable Ca contents in the no LF treatments were significantly higher. Except in the K fertilizer added treatment (i.e. currently adopted 0% biochar + N-P-K-Mg treatment) the exchangeable K content was also significantly higher in the no LF added treatments. However, the reverse was true for the exchangeable Mg contents, where they were lower in the no LF added treatments.

## Discussion

Similar to others who have observed a positive growth response from woody forest plants to charcoal application under field conditions (Chidumayo 1994; Kishimoto and Sugiura 1985; Ishii and Kadoya 1994), application of rubber wood biochar increased the growth of *Hevea* nursery plants of this experiment. In no fertilizer treatments of this study, 2% biochar application increased shoot dry matter in the seedlings by 81% over the control having no biochar (Table 2). However, dry matter accumulation in no LF treatments were far less than LF applied treatments indicating an application of only biochar up to 2% is insufficient to meet the nutritional requirement of the plant for a maximum growth.

Among the fertilizer applied treatments, shoot dry matter accumulation in the 2% biochar + N-Mg applied seedlings and scions have been increased by 29% and 61%, respectively, over the currently adopted N-P-K-Mg LF recommendation. Siregar (2007) observed that charcoal application at rates of 10 or 15% (v/v) would be adequate to improve the availability of soil nutrients, and hence significantly induce a better growth response in two forest plant species, *Acacia mangium* and *Michelia montana*. Gross calculations made based on the bulk volume of biochar and soils used indicated that the 2% (w/w) biochar application rate in our study was approximately 10% on volume basis. But we observed a much low growth without fertilizer. The differences may be attributed to the quality of biochar, plant species and soil properties.

It is puzzling why biochar application did not increase the scion growth in no LF treatments (Table 2) even when some available nutrient contents, as measured at the end of the experiment, were higher compared to the 0% biochar + no LF control (Table 4). Successfully grafted root stock plants were pollarded completely, leaving only 15 cm long snag, in order to induce the scion growth. Dharmakeerthi et al. (2008) observed that after pollarding the top, the need for photosynthates by the root system decreases which result a shedding-off feeder and lateral roots upto about 60% in *Hevea* nursery plants. A new flush of lateral and feeder roots emerges after about 4 to 6 weeks and their growth is controlled, to a great extent, by the photosynthate allocation from the growing scion. Dry matter content of feeder + lateral roots measured at the end of the experiment increased as the leaf dry matter contents increased in the treatments (Table 1) suggesting a higher allocation of photosynthates to the root system in LF added treatments, which also had higher leaf dry weight. Therefore, the resulted weak root system in the no LF treatments probably could have prohibited the shoot benefiting from biochar application. The root:shoot ratio in the no LF treatments was higher compared to the LF added treatments and could be attributed to the adaptation of the root system to uptake more nutrients under a poor nutrient availability in these soils.

For a better plant growth, application of chemical fertilizers was mandatory under the conditions of this experiment. However, compared to the currently recommended N-P-K-Mg treatment, shoot growth in biochar + N-Mg treatments was significantly better. This must be an indication to the effect that either the currently recommended fertilizer levels are not optimum or biochar amendment pushes other unknown mechanisms to improve crop growth.

**Figure 1:**
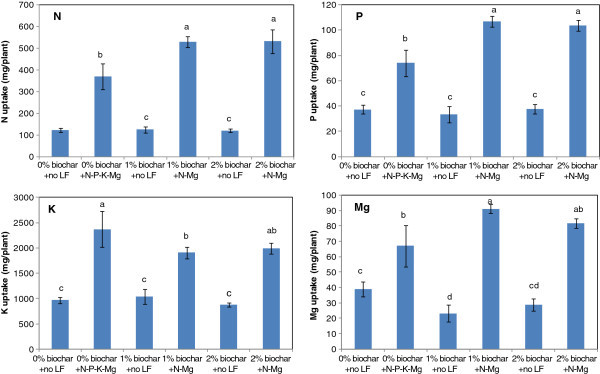
**Uptake of N**, **P**, **K and Mg by the*Hevea*plants at the end of the nursery period as affected by biochar and fertilizer application.** (Error bars indicate the SE of the mean, n = 4; Columns with same letter in each nutrient are not significantly different at p < 0.05).

In order to explore the first possibility, we calculated the amounts of N, P, K and Mg supplied in available form. In terms of chemical fertilizers we have applied 1740 mg of N, 942 mg of P, 822 mg of K and 930 mg of Mg per plant during the 9 month nursery period. These nutrients were applied in liquid form using 100% water soluble sulphate of ammonia, di-ammonium phosphate, sulphate of potash and epsum salt, in split applications at two-week intervals and therefore could be considered as readily available to the plant. Based on the data in Table 1, a plant in the 2% biochar only treatment received 70 mg of available P, 648 mg exchangeable K and 921 mg of exchangeable Mg. In biochar applied treatments, we did not supply P or K as liquid fertilizers. As a result, the highest available P and K contents could be expected in the 0% biochar + N-P-K-Mg treatment whereas the highest available Mg content in 2% biochar + N-Mg treatment. Knicker (2007) argued that nitrogen in biochar may not be available immediately to the plant as N in plant based biochar is usually found in heterocyclic compounds that are part of the biochar matrix. However, presence of biochar in soil could significantly reduce leaching losses of N, P and Mg (Laird et al. 2010b) making them more available to the plant. Therefore, among LF added treatments, N availability could be expected to increase with biochar application rate. We calculated the total nutrient uptake by plants including roots and shoots at the end of the experiment and data indicated that there is a significant increase in N, P and Mg uptake in biochar + N-Mg treatments compared to the 0% biochar + N-P-K-Mg treatment or no LF treatments (Figure 1). Total K uptake was highest in the N-P-K-Mg treatment where K supply was the highest. Although, the increase in Mg uptake in biochar + N-Mg treatments could be attributed to the higher supply of available Mg, high N and P uptake in biochar + N-Mg treatment does not due to high N and P supply. This could in part be due to the reduced leaching losses (Laird et al. 2010b). Moreover, nutrient uptake by plants is controlled not only by nutrient supply but also by the demand of the plant (Devienne-Barret et al. 2000). The demand for nutrients in biochar + N-Mg treatments is the greatest as indicated by their dry matter accumulation. Therefore, high N and P uptake in 2%biochar + N-Mg treatment could also be attributed, in part, to the improved growth.

In order to identify which nutrients had a significant influence on the variations in shoot growth among treatments, a multiple linear regression analysis was conducted where dry matter contents of seedlings and scion shoots were related to the measured leaf nutrient concentrations (Table 5). In both occasions leaf K and Mn concentrations were the only significant variables selected in the final model. Leaf K concentration was the most influential variable, as indicated by the partial R^2^ values, and had a negative effect on plant growth. Along with a decrease in leaf K concentration there was a linear increase in dry matter accumulation. The decrease in plant growth at high leaf K concentrations could be due to the competition between K and Mg at cellular level which involves K induced Mg deficiency (Marschner 1999). A strong antagonism between K and Mg uptake by rubber plants has been recorded by several authors (Weerasuriya and Yogaratnam 1989; Singh et al. 2005) as well. Application of Mg in the form of epsum salt and biochar decreased the K:Mg ratio resulting a decrease in K concentrations in both seedling and scion leaves and an increase of leaf Mg concentrations in the seedling plants.

**Table 5:** **Significant variables selected during the the stepwise regression analysis when shoot dry matter contents were related to the measured leaf nutrient concentrations**, **magnitude of their coefficients and partial R**^**2**^**values**

Growth stage	Independent variable	Dependant variables	Coefficient	R^2^	P value
Before cut-back	Shoot dry matter	Full model		0.596	<0.001
		Intercept	19.7		
		Leaf K concentration	−13.7	0.397	0.002
		Leaf Mn concentration	0.08	0.199	0.043
After cut-back	Shoot dry matter	Full model		0.638	<0.001
		Intercept	9.5		
		Leaf K concentration	– 9.44	0.506	0.016
		Leaf Mn concentration	0.08	0.132	0.012

On the other hand leaf Mn concentration had a positive effect on plant growth suggesting that changes in soil pH bring about by different agro-management practices in these soils could have significant influence on Mn availability and thereby on plant growth. The rubber plant generally grows well in soils with a pH range between 4.0 and 6.5 (Bolton 1964; Krishnakumar and Potty 1992; Priyadarshan 2003). Application of compost and phosphate rock as the basal dressing has increased the soil pH from 4.37 to 5.30 and biochar application further increased the soil pH upto 6.25 (with 1% biochar) and 6.58 (with 2% biochar) immediately after mixing. However, by the end of the 8-month experimental period, pH in no LF treatments ranged from 5.57 to 5.69 with no significant effect observed due to biochar application. Dharmakeerthi et al. (2010) observed that increase in soil pH due to timber mill waste charcoal remained high by about 1 pH units at the end of the nursery period, but without compost and phosphate rock as a basal application. Several others also have observed an increase in soil pH after mixing soils with biochar (Uzoma et al. 2011; Van Zwieten et al. 2010; Chan et al. 2007). When combined with chemical fertilizers, the increase in soil pH has been decreased significantly, almost down to the original soil value (Table 4), again with no significant difference due to biochar application. The decrease in pH could probably be due to the release of protons from NH_4_^+^ during nitrification in sulfate of ammonia added treatments, and even due to reduced K and/or Ca concentrations in the soil due to high plant uptake (Wallace 1994). Zhang et al. (2007) observed that decrease in K and Ca in a rubber growing Ferrosol in China has decreased soil pH significantly. Increased cation uptake can decrease pH in the rhizosphere soils (Riley and Barber 1971). However this finding is contrary to findings of others (Uzoma et al. 2011; Van Zwieten et al. 2010; Chan et al. 2007) who have observed that the increase in soil pH in biochar amended soils remained high till the end of experiment even when fertilizers were applied. The differences could be attributed to the much shorter experimental period of less than 3 months compared to this study, type of fertilizers used or may even be due to the differences in pH buffer capacities. Our findings suggest that application of high alkali rubber wood biochar will not have any lasting effect on pH of this soil when applied together with sulphate of ammonia as the N source. In fact, this is essential given the fact that Mn content in this soil is very low and maintaining an acidic pH is needed to supply Mn requirement of the plant (Kalpage and Silva 1968), if not supplied as a chemical fertilizer.

The second possibility for the better crop growth in biochar + N-Mg applications could be that there are other mechanisms to improve crop growth than nutrient availability. It has been suggested that biochar amendments could lead to change the microbial community in soils, both in structure, abundance and activity (Lehmann et al. 2011). These changes could improve bioavailability of nutrient to the plants and even stimulate the release of plant growth promoting hormones. Blackwell et al. (2010) suggested that benefits from low biochar application rate (~1 Mg ha^-1^) are likely to result from improved crop nutrient and water uptake and crop water supply from increased arbuscular mycorrhizal fungal colonization during dry seasons and in low P soils, rather than through direct nutrient or water supply from biochars. In our study, the nursery was irrigated adequately during non-rainy days to make sure that the plants were not subjected to any moisture limiting situation. An improvement in moisture availability due to biochar could therefore not be the reason for the improved growth in biochar + N-Mg treatments.

It has often been observed that N uptake by plants in biochar amended soils is low and therefore it is required to supplement biochar amended soils with N fertilizer for improved crop growth (Lehmann et al. 2003; Asai et al. 2009). Nitrogen concentrations in leaves of the seedlings and scions, and total N uptake measured at the end of the experiment suggests that for *Hevea* nursery plants, application of biochar did not have any significant negative effect either on N nutritional status (Table 3) or on total N uptake (Figure 1). Leaf P concentrations in both seedlings and scions were not significantly affected by treatments suggesting either P availability in the soil has not been increased or plants were in the optimum P status or higher. In several previous experiments also we have noticed that *Hevea* nursery plants grown in these soils did not respond to currently recommended P levels in these soils. These soils are high in Fe and Al oxides and posses a very high P fixation capacity (Silva et al. 1977). However, significantly high total P uptake in biochar + N-Mg treatments suggests that P acquisition by plants were more efficient in these treatments. Roots of *Hevea* plants can form associations with exotic vesicular arbuscular mycorrhizae species (Jayaratne et al. 1984). The application of biochar could have promoted colonization of mycorrhizae species (Warnock et al. 2007), probably due to changes in N/P ratios in the soil (Miller et al. 2002), resulting an efficient P uptake even in low available P soils.

## Conclusions

Rubber wood biochar produced at high temperature (over 600°C) from firewood used in raw rubber manufacturing factories, could be applied as a soil amendment in *Hevea* young budding nurseries without any negative impacts on plant growth. However, application of rubber wood biochar alone, upto 2% w/w, was not sufficient to improve the nutritional status and growth of the plant. It is essential to apply N and Mg as chemical fertilizers with biochar to produce a plant with better growth. Contrary to some observations on annual crops, application of rubber wood biochar upto 2% (w/w) did not significantly decrease N concentration in leaves. Application of N fertilizer to biochar amended soils did not significantly increase the N status of the plant suggesting that N availability was not the limiting factor for significantly low growth in biochar only treatments. We concluded that growth improvements in biochar + N-Mg treatments were due to decreased K/Mg ratio in leaves, which improved the Mg status of the plant, resulted from Mg fertilizer application and cut down in K fertilizers. Moreover, increases in soil pH due to biochar application could be arrested probably by application of sulfate of ammonia. This should be an essential strategy to adopt in order to supply plant Mn requirement in these soils when amended with high alkali biochar.

The possibility that *Hevea* plants could be grown successfully in rubber wood biochar amended soils with judicious application of chemical fertilizers have several implications on few local and global issues: First, usage of expensive P and K fertilizers in rubber nurseries established in these soils can be cut down completely saving foreign exchange earnings. Second, application of biochar into these highly weathered tropical rubber growing soils will help to arrest fertility degradation or even improve soil fertility. Third, since biochar is believed to be highly resistant to microbial degradation, soil application of rubber wood biochar will further enhance the potential of rubber plantations to sequester atmospheric CO_2_ and contribute towards mitigating climate change issues.

## Methods

A nursery experiment was carried out under field conditions at the Dartonfield Estate (N 6° 30.278^′^ and E 80° 10.091^′^) in Agalawatta of the Rubber Research Institute of Sri Lanka. Plants were raised in black polyethelene bags filled with soil or soil amended with biochar. Soils from the *Agalawatta* series (Typic Hapludults) were collected from the surface layer (0 – 15 cm depth) of the nursery site and biochar were produced at the institute. During the 8-month long nursery period, growth and nutritional status of the plants were assessed at two stages, around grafting and when the plants are ready for field planting.

### Production of biochar

Retort method was adopted to make biochar from rubber wood of the trunk of uprooted mature rubber plants. These logs are used as a source of firewood in raw rubber manufacturing factories in Sri Lanka. The air-dried logs (~18% moisture w/w) were chopped to about 3 – 6 cm thick and 40 cm long pieces and packed tightly into a 30 cm diameter × 45 cm height metal container having six 1-cm diameter holes at the bottom. The container was closed with the lid, placed in the middle of firewood inside a furnace of a ribbed smoke sheet rubber manufacturing factory. The firewood in the furnace was then fired. Within 45 minutes the temperature inside the furnace rose to about 600°C and a temperature over 600°C was maintained inside the furnace by adding firewood periodically. When the syngas production from the material inside the retort was exhausted, around 3 and Â½ h, it was retrieved from the furnace and the produced biochar was quenched with water immediately to prevent rapid oxidation and self-ignition. Three such batches of biochar were made and composited. About 25-28% dry biomass had been converted into biochar in this method. Later this biochar was air-dried for 6 days, ground to pass through a 2-mm sieve and used for the experiment.

### Nursery practices and treatments

Soils were sieved through a 1 cm mesh to remove large lateritic gravel, mixed with 0, 1 and 2% (w/w) rubber wood biochar and filled into gauge 300 polyethelene bags having lay-flat dimensions of 15 cm diameter and 37 cm height. At bag filling, 50 g of compost made out of animal manure and plant residues and 50 g of *Eppawala* rock phosphate were also added to each bag as a basal application. After two weeks, a rubber seedling showing very high vigor, based on early germination and growth rate in the germination bed, which was established using fresh seeds collected at the beginning of the seed fall season, was transplanted in each bag.

Four weeks after transplanting, application of chemical fertilizers, in liquid form, into treatments was started and carried out at two-week intervals. Half of the bags without biochar were treated with currently recommended N-P-K-Mg liquid fertilizer (LF) formulation (Samarappuli 2001). As Dharmakeerthi et al. (2010) have observed in a previous study conducted using same soil and commercially available charcoal, that the P and K uptakes by the nursery plants were more than the critical level while N and Mg uptakes were very low, and as rubber plants are also characterized for its K and Mg antagonism (Weerasuriya and Yogaratnam 1989), where high soil K levels affect Mg uptake, half of the biochar applied bags were treated only with N and Mg in the currently recommended LF formulation. The remaining bags incorporated with 1 and 2% biochar were not supplied with any LF. This has resulted 6 treatment combinations (Table 6). Each treatment had 10 single plant replicates and they were arranged in a randomized complete block design in a nursery established under field conditions in February 2010. In order to prevent root growth into the ground through holes at the bottom of the bags, a black polyethelene sheet was placed underneath. During the non rainy days, plants were irrigated manually using a watering can, once in every other day, until the soil is saturated.

**Table 6:** **Summary description of the application of compost**, **phosphate rock**, **liquid fertilizer** (**LF**) **and biochar into different treatments**

Trt.No.	Basal applicationÂ§		Treatment					Abbreviation
	Compost	Phosphate rock	Liquid fertilizer†				Biochar‡	
			N	P	K	Mg		
1	50 g	50 g	-	-	-	-	0%	0% biochar + no LF
2	50 g	50 g	√	√	√	√	0%	0% biochar + N-P-K-Mg
3	50 g	50 g	-	-	-	-	1%	1% biochar + no LF
4	50 g	50 g	√	-	-	√	1%	1% biochar + N-Mg
5	50 g	50 g	-	-	-	-	2%	2% biochar + no LF
6	50 g	50 g	√	-	-	√	2%	2% biochar + N-Mg

Â§ Compost and phosphate rock, 50 g each, were applied into a polybag at bag filling stage.

† LF were applied (√) at two-week intervals at rates recommended by the Rubber Research Institute of Sri Lanka (Samarappuli, 2001). N, P, K and Mg were supplied as sulfate of ammonia, di-ammonium phosphate, sulfate of potash and commercial epsom salt, respectively.

‡ Biochar (w/w) were mixed with soil, together with compost and phosphate rock, at bag filling stage.

The nursery was managed as per the recommendations of the Rubber Research Institute of Sri Lanka (Tillekeratne and Nugawela 2001) in obtaining clonal rubber plants. Thereby, when the seedling plants (root stock) were 14 weeks old (grafting stage), base of their stems were grafted with a bud patch obtained from the clone RRIC 121. Four weeks thereafter, the successfully grafted seedling plants were pollarded (cut-back stage) leaving a 15-cm snag above the bud patch and this facilitated the sprouting of the bud and growth of the desired clonal plant (scion). The scion was allowed to grow for 12 weeks before destructively sampled for measurements of dry matter in different components and total nutrient uptake.

### Plant analyses

Above ground dry matter contents of the seedling plants at the cut-back stage and above and below ground dry matter contents of the scion at 12 weeks after cut-back stage were determined. Leaves were separated from the rest of the above ground parts. The root system was washed gently under running water over a 0.5 mm sieve and the adhering soil and dust particles were carefully removed. Roots initiating from the tap roots were considered as lateral roots, and fibrous roots arising from laterals were considered as feeder roots. All decaying brown colored roots were considered as dead or near dead and were removed prior to determinations.

Plant samples were dried at 70°C to a constant weight and the dry matter contents were recorded. All leaves in a plant were composited to determine the leaf nutrient concentrations at the two growth stages. Macro nutrients (N, P, K, Mg and Ca) were determined by digesting ground leaf samples in Conc. H_2_SO_4_ / 30% H_2_O_2_ (Wolf 1982) while micro nutrients (Fe, Zn and Mn) by digesting leaf samples in Conc. HClO_4_ and HNO_3_ / 50% HCl mixture (PCARR 1980).

### Soil and biochar analyses

Soil samples, collected at the beginning and end of the experiment were air-dried and sieved through a 2-mm sieve. Samples of biochar were dried at 105°C to a constant weight prior to analysis. In both sets of soil and biochar samples, following analyses were conducted: available P in 1 M NH_4_F / 0.5 M HCl (pH 1.8) extraction (Bray and Kurtz 1945), 1 M NH_4_OAc (pH 7) exchangeable K, Mg and Ca contents (Hesse 1971) and total N, P, K, Mg and Ca contents as described by (Singh and Ratnasingam 1971), cation exchange capacity by replacing cations with 1 M NH_4_OAc (pH 7) and extracting with 1 M KCl (Handershot et al. 1993). pH in biochar were determined using a 1:20 (water) suspension as described by (Rajkovich et al. 2011) while soil pH was determined in a 1:2.5 (water) suspension (Rowell 1994) using a glass electrode in Jenway 3510 pH meter. The ash contents in soil and biochar samples were determined by ASTM D2974 1988 method as described by Karam (1993). Walkey and Black organic C content (Nelson and Sommers 1982) and particle size distribution according to the pipette method (Day 1965) were also determined in soil samples.

Nutrient concentrations in soil, plant and biochar extracts were determined using SKALAR San++ segmented flow analyzer and GBC 9003 atomic absorption spectrophotometer.

### Statistical analyses

Analysis of variance was conducted using PROC GLM program of the SAS software package (SAS Institute Inc. 2004). Treatment means were separated using the Duncan’s multiple range test. Multiple linear regression analysis, relating shoot dry matter contents to leaf nutrient concentrations, was conducted by stepwise linear regression procedure in the PROC REG program of the SAS software package.

## Authors’ information

RSD is a Senior Soil Scientist at the Rubber Research Institute of Sri Lanka. His research program is focused on the impact of biochar amendments on soil quality, agricultural productivity and carbon sequestration. He also works on small scale biochar production technologies. RSD received his PhD from the University of Guelph, Canada in 2002. JASC and VUE are Experimental Officers attached to the RSD’s research group. VUE graduated from the Open University, Sri Lanka. Their main interests are to develop analytical techniques to characterize biochar and to study nutritional requirement of the rubber plant.
